# Production of 3-Hydroxypropionic Acid from Renewable Substrates by Metabolically Engineered Microorganisms: A Review

**DOI:** 10.3390/molecules28041888

**Published:** 2023-02-16

**Authors:** Xiaodi Wang, Zhenzhen Cui, Xi Sun, Zhiwen Wang, Tao Chen

**Affiliations:** 1 Department of Biochemical Engineering, School of Chemical Engineering and Technology, Tianjin University, Tianjin 300350, China; 2 Frontier Science Center for Synthetic Biology and Key Laboratory of Systems Bioengineering (MOE), School of Chemical Engineering and Technology, Tianjin University, Tianjin 300350, China

**Keywords:** 3-hydroxypropionic acid, carbon sources, metabolic engineering, synthetic biology

## Abstract

3-Hydroxypropionic acid (3-HP) is a platform chemical with a wide range of existing and potential applications, including the production of poly(3-hydroxypropionate) (P-3HP), a biodegradable plastic. The microbial synthesis of 3-HP has attracted significant attention in recent years due to its green and sustainable properties. In this paper, we provide an overview of the microbial synthesis of 3-HP from four major aspects, including the main 3-HP biosynthesis pathways and chassis strains used for the construction of microbial cell factories, the major carbon sources used for 3-HP production, and fermentation processes. Recent advances in the biosynthesis of 3-HP and related metabolic engineering strategies are also summarized. Finally, this article provides insights into the future direction of 3-HP biosynthesis.

## 1. Introduction

3-Hydroxypropionic acid (3-HP) is an important bulk chemical with a wide range of applications in chemical synthesis. Since it contains two reactive groups—hydroxyl and carboxyl—one of the important applications of 3-HP is its polymerization to yield poly(3-hydroxypropionate) (P-3HP). Due to its biodegradability and biocompatibility, P-3HP is one of the widely studied substitutes for petrochemical plastics, with great application value and development prospects [1]. In addition, 3-HP can undergo redox reactions to produce 1,3-propanediol (1,3-PDO), acrylic acid, malonic acid, acrylamide, acrylonitrile, etc. [2], which are widely used in the production of adhesives, plastic packaging, fibers, and cleaning agents. In particular, acrylic acid occupies the highest market, with a value of USD 12 billion in 2020, which is projected to be around USD 19.2 billion by 2030 [3,4]. Due to the significant commercial application potential of 3-HP, the market value and size are estimated to be more than USD 10 billion/year and 3.6 million tons/year, respectively [3].Currently, 3-HP production is mainly based on chemical synthesis from fossil feedstocks, but chemical production is costly and environmentally unsafe. 3-HP was listed as one of the most promising bulk chemicals that can be obtained from renewable materials by the U.S. Department of Energy [5,6], which can be achieved using engineered microorganisms. In the past few years, several industrial parties, such as OPX Biotechnologies, Cargill, Novozymes, and BASF, have developed pilot plants for the bio-based production of 3-HP and its chemical conversion to AA [4,7,8,9]. A series of genetically engineered microbial cell factories to convert renewable substances into 3-HP have been constructed via different biosynthetic pathways [3,10,11]. However, microbial 3-HP synthesis processes still has a certain distance to travel meet the market demand. 

The most important strategy for developing microbial 3-HP synthesis processes is the selection of suitable microbial chassis strains, effective metabolic pathways and economical substrate, but the effects of fermentation processes on overall process economics should also not be ignored. In this paper, we give a brief overview of the main 3-HP biosynthesis pathways that have been used for the development of microbial cell factories. Notably, we summarize the excellent cases of microbial 3-HP production from both chassis and substrate aspects. In this way, readers will have a clearer understanding of the current status of 3-HP production, i.e., what are the 3-HP production pathways, chassis and substrates, and researchers who want to establish 3-HP microbial cell factories will be able to better select and combine them. In terms of chassis, we summarize the excellent chassis used for 3-HP production and introduce their characteristics in the production process. In terms of substrates, we not only summarize the excellent cases of common carbon sources (glycerol and glucose) in 3-HP production, but also outline the cases of other emerging carbon sources, such as xylose, acetate, fatty acids, ethanol, and CO_2_.

## 2. Main 3-HP Biosynthesis Pathways for the Construction of Microbial Cell Factories

**Figure 1:**
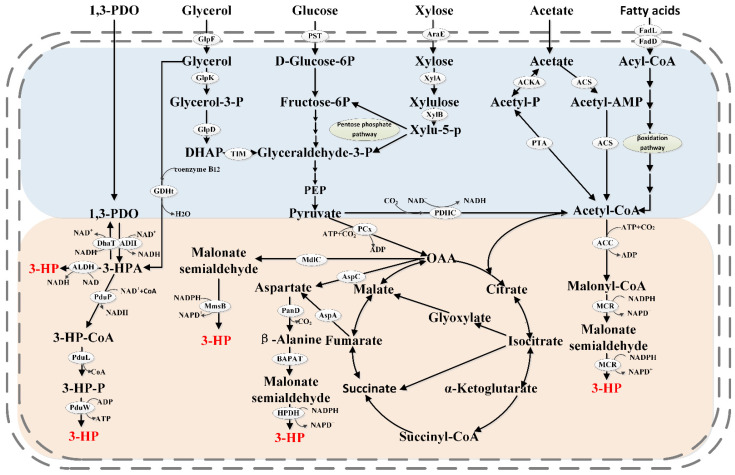
Main pathways and key enzymes for 3-HP biosynthesis from renewable substrates. Blue module: assimilation pathways of different renewable substrates; orange module: various 3-HP biosynthesis pathways. 1,3-PDO, 1,3-propanediol; 3-HPA, 3-hydroxypropionaldehyde; 3-HP-P, 3-hydroxypropionyl-phosphate; ACC, acetyl-CoA carboxylase; ACKA, acetate kinase; ACS, acetyl-CoA synthase; ADH, alcohol dehydrogenase; ALDH, aldehyde dehydrogenase; AraE, major facilitator superfamily transporter; AspA, aspartate ammonia-lyase; AspC, aspartate aminotransferase; BAPAT, β-alanine pyruvate transaminase; DHAP, dihydroxyacetone phosphate; DhaT, 1,3-propanediol dehydrogenase; FadD, long-chain acyl-CoA synthetase; FadL, long-chain fatty acid transport protein; GDHt, glycerol dehydratase; GlpD, glycerol-3-phosphate dehydrogenase; GlpF, glycerol uptake facilitator protein; GlpK, glycerol kinase; HPDH, 3-hydroxypropanoic acid dehydrogenase; MCR, malonyl-CoA reductase; MdlC, benzoylformate decarboxylase; MmsB, 3-hydroxyisobutyrate dehydrogenase; PanD, aspartate-α-decarboxylase; PCx, pyruvate carboxylase; PDHC, pyruvate dehydrogenase; PduL, phosphotransferase; PduP, propionaldehyde dehydrogenase; PduW, propionic acid kinase; PTA, phosphate acetyltransferase; PTS, phosphotransferase system; XylA, xylose isomerase ; XylB, xylulokinase.

Many 3-HP synthesis pathways have been identified, and these have been described in detail in excellent reviews by Kumar [12] and de Fouchécour [11]. The main pathways that have been used for the construction of 3-HP cell factories are shown in Figure 1. Based on the key intermediate metabolites, they are termed the glycerol pathway (including the coenzyme A-dependent pathway and coenzyme A-independent pathway), malonyl coenzyme A pathway, β-alanine pathway, and 1,3-PDO pathway. Microbial cell factories for the production of 3-HP via these pathways have all been developed over the years. Notably, the glycerol and 1,3-PDO pathways have a shorter conversion path from substrate to product, which gives them significant advantages in 3-HP production compared to the longer malonyl-CoA and β-alanine pathways. 

### 2.1. Glycerol Pathway

#### 2.1.1. Glycerol Oxidation via the Coenzyme A-Dependent Pathway

The coenzyme A-dependent pathway was discovered in *Lactobacilli* spp., which can naturally metabolize glycerol to synthesize 3-HP [13]. In *Lactobacillus reuteri*, glycerol is converted into 3-hydroxypropionaldehyde (3-HPA) by coenzyme B_12_-dependent glycerol dehydratase (PduCDE), after which propionaldehyde dehydrogenase (PduP) catalyzes the ligation of 3-HPA to coenzyme A, yielding 3-HP-CoA. The final conversion of 3-HP-CoA to 3-HP is catalyzed by phosphotransferase (PduL) and propionic acid kinase (PduW), and the whole production process is accompanied by the generation of one molecule of ATP and one molecule of NADH [14,15,16].

#### 2.1.2. Glycerol Oxidation via the Coenzyme A-Independent Pathway

The coenzyme A-independent pathway requires only two reactions to produce 3-HP from glycerol. Similar to the coenzyme A-dependent pathway, glycerol is dehydrated by coenzyme B_12_-dependent glycerol dehydratase (GDHt) to produce 3-HPA, but then it is oxidized into 3-HP by only one enzyme, aldehyde dehydrogenase (ALDH), instead of three enzymes. Due to the short production pathway and few metabolic intermediates, this is the most widely studied pathway for the microbial production of 3-HP from glycerol. However, the low enzyme activity of natural ALDH, together with the imbalance of expression between ALDH and GDHt, are the main limiting factors of the coenzyme A-independent pathway.

### 2.2. Malonyl-CoA Pathway

The 3-hydroxypropionic acid cycle was discovered in *Chloroflexus aurantiacus* as an autotrophic carbon fixation pathway [17]. In subsequent studies, researchers used a part of the reactions from the 3-hydroxypropionic acid cycle (from acetyl-CoA to 3-HP) for 3-HP production and named it the malonyl-CoA pathway [18]. In the malonyl-CoA pathway, acetyl-CoA is converted into malonyl-CoA by acetyl-CoA carboxylase (ACC), which fixes one molecule of CO_2_ at the expense of one molecule of ATP, after which the malonyl-CoA is converted into 3-HP by a two-step reaction catalyzed by NADPH-dependent malonyl-CoA reductase (MCR). The MCR consists of two different functional domains: MCR-C catalyzes the reduction of malonyl-CoA to malonyl semialdehyde (MSA) and MCR-N catalyzes the reduction of MSA to 3-HP, and the overall reaction consumes a total of two molecules of NADPH. In addition to glucose, it is also possible to use ethanol, fatty acids, acetic acid, and other less common carbon sources to produce 3-HP via the malonyl-CoA pathway, since acetyl-CoA is a common metabolic intermediate.

### 2.3. β-Alanine Pathway

β-alanine can be formed by the transamination or decarboxylation of various amino acids, such as arginine, lysine, and aspartic acid, which are widely available metabolites in microorganisms [19]. At present, β-alanine is usually synthesized from aspartic acid using aspartate decarboxylase (PanD), while aspartic acid can be synthesized from fumarate or oxaloacetate by transamination, which is used for 3-HP synthesis via the β-alanine pathway. The β-alanine pathway was first constructed in *E. coli* by Cargill [20]. In this pathway, β-alanine is converted into MSA by β-alanine pyruvate transaminase (BAPAT), after which MSA is reduced to 3-HP by NADPH-dependent 3-hydroxypropanoic acid dehydrogenase (HPDH) [21]. In subsequent studies, γ-aminobutyric acid transaminase (GABT) was also used for the conversion of β-alanine to MSA [22]. 

### 2.4. 1,3-Propanediol Pathway

In *Acetobacter*, alcohols are selectively oxidized to the corresponding aldehydes and ketones by alcohol dehydrogenase (ADH), and the aldehydes are further oxidized to the corresponding carboxylic acids by aldehyde dehydrogenase (ALDH) [23]. *Gluconobacter oxydans* has a native capacity to convert 1,3-PDO to 3-HP. Further, a recent study revealed the relevant enzymes catalyzing this process by UV mutagenesis and transcriptome analysis techniques in *G. oxydans* [24]. In this pathway, 1,3-PDO is converted to 3-HPA by ADH, after which 3-HPA is converted to 3-HP by ALDH. It was reported that ALDH is the rate-limiting enzyme of this production process [24], so balancing the activity of both enzymes is an effective strategy to increase the titer of 3-HP.

### 2.5. Other Pathways

The lactate pathway and the oxaloacetate pathway have also been successfully constructed to biosynthesis 3-HP. It was found that D-lactate and 3-HP are hydroxyl position isomers by a chemical structural formula analysis, and the synthesis of 3-HP from D-lactate can be achieved by a hydroxyl shift reaction. However, the lactate pathway is thermodynamically infeasible because the overall standard Gibbs free energy change is positive [12]. Wang et al. [25] introduced the lactate pathway into a lactate-producing *E. coli* chassis, but less than 5% of lactate could be converted to 3-HP, and the titer of the engineered *E. coli* was about 2.0 g/L in fed-batch fermentation. In addition, the similar chemical structure of lactate and 3-HP contributed to the difficulty of downstream isolation. Therefore, the lactate pathway is an unwelcome option in 3-HP biosynthesis.

Due to the long β-alanine pathway, the oxaloacetate pathway was recently constructed as a simpler alternative. In the oxaloacetate pathway, benzoylformate decarboxylase (MdlC) catalyzes the direct conversion of oxaloacetate to MSA, avoiding the three-step reaction found in the original β-alanine pathway [26]. Notably, the oxaloacetate pathway (ΔG° = −34.7 kJ/mol) demonstrated to be thermodynamically more favorable and feasible than the malonyl-CoA pathway (ΔG° = −14.5 kJ/mol) [26]. Therefore, this simplified pathway provides an attractive alternative to the existing 3-HP synthesis routes. At present, the oxaloacetate pathway has only been constructed in *Saccharomyces cerevisiae*, and the genetically engineered *S. cerevisiae* showed good potential for 3-HP production, reaching a titer of 18.1 g/L. Further, it is still necessary to conduct additional studies on the systematic comparison of key enzymes from different sources and the construction of the oxaloacetate pathway in other chassis.

## 3. Chassis Strains for 3-HP Production

The chassis cells that have been engineered for the biosynthesis of 3-HP include *Klebsiella pneumoniae*, *Escherichia coli*, *Corynebacterium glutamicum*, *Saccharomyces cerevisiae*, *Halomonas bluephagenesis*, and *Pseudomonas denitrificans*. Among them, *Klebsiella pneumoniae*, *Halomonas bluephagenesis*, and *Pseudomonas denitrificans* exhibited the most promising potential for 3-HP production. Representative studies on the modification of different chassis strains for the synthesis of 3-HP are summarized in Table 1.

### 3.1. Klebsiella pneumoniae

*K. pneumoniae* is able to grow rapidly with glycerol as the only carbon source under both aerobic and anerobic conditions. Two groups’ regulons involved in glycerol metabolism have been identified in *K. pneumoniae*. Under aerobic conditions, the glp regulon (encodes a series of enzymes required for the conversion of glycerol to DHAP via sn-glycerol-3-phosphate) acts to oxidize and catabolize glycerol, mainly via the Embden–Meyerhof–Parnas (EMP) pathway. Under anaerobic conditions, instead of the glp regulon, the dha regulon (encodes a series of enzymes needed for the anaerobic growth on glycerol in the absence of exogenous electron acceptors) acts to oxidize glycerol via the EMP pathway, as well as to dehydrate glycerol to 3-HPA via GDHt (encoded by *dhaB*), which can be converted to 3-HP [54]. Moreover, it possesses native genes associated with both the coenzyme A-dependent and coenzyme A-independent 3-HP biosynthetic pathways. However, the studies on 3-HP production in *K. pneumoniae* were mainly focused on glycerol oxidation via the coenzyme A-independent pathway. Coenzyme B_12_, which is an expensive but important co-factor for the dehydration of glycerol to produce 3-HPA, is naturally produced by *K. pneumoniae* under anoxic conditions. Interestingly, the synthesis of coenzyme B_12_ and the transcription of *dhaB* showed a decreasing trend with increasing aeration, while the regeneration of NAD^+^ (a co-factor of ALDH) through the electron transport chain required oxygen [55]. Therefore, the degree of aeration has a significant effect on the production of 3-HP in *K. pneumoniae*. Ashok et al. [56] investigated the production of 3-HP by engineered *K. pneumoniae* at different aeration levels. Under high aeration and anaerobic conditions, only a small amount of 3-HP was produced from glycerol. High titers of 3-HP were only produced under microaerophilic conditions with constant dissolved oxygen (DO) at 5%. At present, the highest 3-HP titer in *K. pneumoniae*, which reached 102.61 g/L, was achieved by overexpressing PuuC through three tandem repeats of the tac promoter [27]. Furthermore, this study stressed the importance of maintaining active cell growth, which drives lactate back to pyruvate in the late stages of fermentation without blocking the lactate synthesis pathway, and the yield of 3-HP was further increased to 0.86 g/g [27]. However, *K. pneumoniae* is a pathogenic bacterium that can cause multiple infections, leading to biosafety issues when using it on an industrial scale [57]. Recently, *Klebsiella* spp. *AA405* was identified as a novel strain with low levels of virulence factors and half the endotoxin of *E. coli*, and it is expected to become an emerging 3-HP producing chassis in the future [58]. 

### 3.2. Escherichia coli

*E. coli* is a model organism with the advantages of a clear genetic background, easy modification, broad substrate spectrum, simple growth conditions, and mature means of genetic manipulation, which have made it the most widely used host organism in biotechnology. However, *E. coli* is less tolerant to 3-HP than other microorganism, growing normally only in neutral basic salt media with less than 18 g/L 3-HP, and failing to grow at all when the 3-HP concentration is higher than 45 g/L [33]. A highly 3-HP-tolerant strain of *E. coli* was developed through adaptive laboratory evolution (ALE), and after the reversion of mutation in glycerol kinase GlpK, the strain produced a significantly higher 3-HP titer (63.05 g/L) than the parental strain (49.09 g/L) in bioreactor culture. Further genomic analysis revealed that mutations in the *yieP* gene confer 3-HP tolerance in *E. coli* [33]. To date, three 3-HP synthesis pathways have been successfully constructed in *E. coli*. There is no endogenous pathway in *E. coli* that catalyzes the conversion of glycerol to 3-HPA, and *E. coli* cannot naturally produce coenzyme B_12_. Therefore, the production of 3-HP via the glycerol pathway in *E. coli* requires the introduction of heterologous GDHt and the exogenous addition of expensive coenzyme B_12_, which undoubtedly affects the economics of the production process. By contrast, the production of 3-HP via the malonyl-CoA pathway and the β-alanine pathway does not require the involvement of coenzyme B_12_, which provides a promising alternative for the production of 3-HP when using *E. coli* as the chassis cell [57]. Nevertheless, the highest 3-HP titers reported in engineered *E. coli* via the β-alanine pathway (31.1 g/L) [38] and the malonyl-CoA pathway (52 g/L) [36] were much lower than that obtained via the glycerol pathway (76.2 g/L) [31]. In the future, the factors limiting 3-HP production in *E. coli* via the remaining two pathways, such as the imbalance or low activity of key enzymes in the production pathway and a tightly regulated accumulation of metabolic intermediates, need to be further addressed. In addition, the study that obtained a titer of 76.2 g/L clearly showed that the titer, yield, and productivity were significantly dependent on the culture conditions, including dissolved oxygen levels and feeding strategies [31]. It is an important task in metabolic engineering for the fermentation condition to be optimized for each engineered strain, as the optimal condition for the selected strain is not guaranteed to be the best for another engineered strain. 

### 3.3. Yeasts

Yeasts have become popular chassis strains because they are highly acid tolerant, which abrogates the need for the exogenous addition of an alkaline titrant in the production of 3-HP. As a consequence, the downstream recovery process is simplified, with the end product being 3-HP instead of 3-hydroxypropionate when using yeast as the chassis. However, yeasts are eukaryotic organisms whose metabolic network is more complex than that of bacteria, and the 3-HP titers obtained in yeast to date remain low. In recent studies, the malonyl-CoA pathway and the β-alanine pathway have been successfully constructed in different yeasts.

#### 3.3.1. *Saccharomyces cerevisiae*

A report showed that *S. cerevisiae* could grow in the presence of 25 g/L of 3-HP with half of its μ_max_. In another study, an *S. cerevisiae* strain, which could tolerate 50 g/L of 3-HP at pH 3.5, was obtained by ALE. Further, a genome-wide analysis indicated that high concentrations of 3-HP are converted to 3-HPA via aldehyde dehydrogenase, which in turn leads to cytotoxicity. To compensate for this, *S. cerevisiae* used a glutathione-dependent formaldehyde detoxification mechanism to achieve higher tolerance to 3-HP [59]. Borodina’s team reported an engineered *S. cerevisiae* with an introduced β-alanine pathway, which produced a 3-HP titer of 13.7 g/L with a 0.14 Cmol/Cmol yield from glucose in controlled fed-batch fermentation [60]. Subsequently, another team examined the effect of changing the culture parameters on the microbial production performance based on the strains mentioned above, and the highest 3-HP titer reached 25 g/L with a yield of 25.6% Cmol/Cmol under P-limited conditions [45]. In addition, the oxaloacetate pathway for 3-HP biosynthesis was also developed in *S. cerevisiae*, and a dephosphorylation regulation strategy was developed to increase the ATP supply. As a result, the engineered strain produced 18.1 g/L of 3-HP in a 5 L bioreactor [26]. Currently, the production advantages exhibited by *S. cerevisiae* are not obvious compared to other 3-HP producing strains.

#### 3.3.2. *Pichia pastoris*

An interesting trait of *P. pastoris* is that it is a Crabtree-negative yeast and can grow well on glycerol, but it does not synthesize coenzyme B_12_. A new hotspot is the construction of *P. pastoris* cell factory for 3-HP production from glycerol via the malonyl-CoA pathway. Among yeasts using glycerol as a substrate, an engineered strain of *P. pastoris* achieved the highest 3-HP titer and productivity reported so far, reaching 37.05 g/L and 0.71 g/(L·h), respectively [40]. This demonstrates that glycerol may be a potential substrate for the malonyl-CoA pathway.

#### 3.3.3. Other Yeast Species 

Compared with *S. cerevisiae*, *Schizosaccharomyces pombe* showed better 3-HP tolerance, as its μ_max_ only decreased from 0.3 h^−1^ to 0.25 h^−1^ in the presence of 50 g/L of 3-HP, which makes it a useful host for 3-HP production [61]. Recently, a strain of *Debaryomyces hansenii* that can naturally convert propionic acid to 3-HP was screened from orchard soil, and it was found to be able to tolerate high concentrations of propionic acid. Moreover, this strain produced 62.42 g/L of 3-HP in rich medium containing 30 g/L of glucose and 10 g/L of propionic acid, indicating that it has promising application potential in the biosynthesis of 3-HP [43].

### 3.4. Corynebacterium glutamicum

*Corynebacterium glutamicum* is known for its ability to produce high titers of amino acids, and it is considered a highly promising strain for 3-HP production due to its broad substrate spectrum and high organic acid production capacity. Both the glycerol pathway and the malonyl-CoA pathway have been successfully constructed in *C. glutamicum*. The highest titer of 3-HP in *C. glutamicum* was obtained through the glycerol pathway, which reached 62.6 g/L using sugar as the substrate, and the maximum yield from glucose reached 0.51 g/g by enhancing the flux towards 3-HP [46]. Recently, it was reported that 166.6 g/L of β-alanine was produced in fed-batch fermentation using engineered *C. glutamicum* [62]. As it is the direct precursor in the β-alanine pathway for 3-HP biosynthesis, there is great potential for 3-HP production in *C. glutamicum* via the β-alanine pathway.

### 3.5. Halomonas bluephagenesis

The extremophilic *Halomonas* spp. are noted for glowing well at alkaline pH and high salt concentrations [63]. These unique growing conditions offer several advantages, enabling production under open non-sterile conditions and providing strain tolerance to high concentrations of 3-hydroxypropionate. However, the genetic background of *Halomonas* spp. is not as clear as that of typical model organisms, which makes it inconvenient to optimize the metabolic network when constructing microbial cell factories. Another problem is the higher cost of 3-HP separation from the fermentation broth when using *Halomonas* spp. as the production chassis, as the product is accumulated in the form of its salt and not the free acid. Currently, only the 1,3-PDO pathway has been constructed in *Halomonas bluephagenesis*. A two-stage production process with glucose as the growth stage substrate and 1,3-PDO as the production stage substrate was reported, in which the engineered *H. bluephagenesis* could accumulate a 3-HP titer of up to 154 g/L, with a high yield of 0.93 g of 3-HP/g 1,3-PDO, which is the highest titer of 3-HP obtained by biological methods to date [48]. 

### 3.6. Pseudomonas denitrificans

*Pseudomonas denitrificans* is considered an ideal host for the production of 3-HP from glycerol under aerobic conditions, since coenzyme B_12_ is naturally synthesized under aerobic rather than anoxic conditions in this chassis and there is no need to worry about the conflict between coenzyme B_12_ synthesis and NADH regeneration [64]. However, researchers found that the amount of coenzyme B_12_ produced by *P. denitrificans* cell factories was not sufficient enough to synthesize 3-HP with high titers from glycerol. According to research reported by the Park team, the 3-HP production of recombinant *P. denitrificans* was dramatically increased from 20.8 g/L to ~100 g/L by enhancing the synthesis of coenzyme B_12_, and the team ultimately achieved a 3-HP titer of 102 g/L and a productivity of 2.5 g/(L·h) [50,65]. Significantly, the productivity of recombinant *P. denitrificans* has already met the minimum needs of industrial production in fed-batch fermentation. The malonyl-CoA pathway was also constructed in *P. denitrificans*, but only mg levels of 3-HP were produced [66]. Unfortunately, *P. denitrificans* contain natural 3-HP degradation pathways, which can grow on 3-HP or degrade 3-HP under non-growth conditions. There is a need to further identify and remove the 3-HP assimilation pathway to develop more efficient *P. denitrificans* biorefineries.

### 3.7. Other Chassis Strains

In recent studies, *Bacillus subtilis* and *Pseudomonas asiatica* have also been used as chassis strains for the synthesis of 3-HP. Although *B. subtilis* demonstrated the ability to rapidly take up glycerol and grow well on glycerol as the substrate, it is limited by its inability to synthesize coenzyme B_12_. The engineered *B. subtilis*, with an introduced glycerol synthesis pathway and modulated expression of glycerol assimilation genes, produced 10 g/L of 3-HP in shake flask fermentation using glycerol as the substrate [49]. Similar to *P. denitrificans*, *P. asiatica* C1 also naturally synthesizes coenzyme B_12_ under aerobic conditions. *P. asiatica* C1 was engineered for 3-HP production by overexpressing genes related to the coenzyme A-independent synthesis pathway, disrupting genes responsible for 3-HP degradation, and promoting glycerol assimilation, which resulted in a 3-HP titer of 63 g/L and a yield of 0.99 mol/mol of glycerol in fed-batch fermentation [51]. 

With the development of third-generation microbial refineries, photosynthetic microorganisms such as cyanobacteria are being engineered for the production of chemicals directly from CO_2_. However, a pathway for synthesizing glycerol and converting it into 3-HP is not available in cyanobacteria. At present, only the malonyl-CoA pathway and the β-alanine pathway have been reported in cyanobacteria. Zhang et al. [52] constructed an optimized malonyl-CoA pathway in *Synechocystis* sp. PCC 6803 and produced 837.18 mg/L of 3-HP. Another excellent production strategy was to construct a microbial consortium consisting of cyanobacteria and *E. coli*, in which engineered *Synechococcus elongatus* was responsible for the synthesis of sucrose from CO_2_ and engineered *E. coli* further synthesized 3-HP from sucrose. Unfortunately, the 3-HP titers obtained using cyanobacteria remain very poor.

## 4. Renewable Carbon Sources for the Biosynthesis of 3-HP

A wide range of renewable feedstocks have been investigated as substrates for the microbial production of 3-HP. While glycerol and glucose were studied in the most detail, emerging carbon sources, such as xylose, acetic acid, fatty acids (FAs), ethanol, and even CO_2_, have also been used for 3-HP production due to their environmental friendliness and higher theoretical conversion rates. Representative studies using engineered strains for 3-HP production from various carbon sources are summarized in Table 1. 

### 4.1. Microbial Production of 3-HP from Glucose or Xylose

Most commercially available glucose is produced by hydrolyzing corn starch, which makes it a cheap and abundant resource. Most microorganisms can utilize glucose for the growth and production of various fermentation products, and the conversion pathway from glucose to 3-HP is redox neutral [11]. Therefore, the production of 3-HP from glucose as a common carbon source has been widely studied. In recent years, to avoid competition with humans for food, researchers have developed microbial cell factories that use lignocellulosic biomass (LCB) derivatives. The fermentable sugars formed by the hydrolysis of LCB are mainly glucose and xylose [67], so it is necessary to construct microbial cell factories for the synthesis of 3-HP that use xylose as the substrate. In contrast to glucose, many microorganisms are unable to use xylose naturally, which requires the introduction of heterologous xylose metabolic pathways. Moreover, recombinant strains tend not to grow well on xylose, which makes it more attractive to produce 3-HP using both glucose and xylose as mixed substrates.

#### 4.1.1. Biosynthesis of 3-HP via the Glycerol Pathway

Seo’s team developed a production strategy for 3-HP synthesis via the glycerol pathway by co-utilizing glucose and xylose as mixed carbon sources in *E. coli*. First, the conversion of glucose and xylose to glycerol was enhanced by an overexpression of *S. cerevisiae*-derived glycerol-3-phosphate dehydrogenase (GPD1) and glycerol 3-phosphatase (GPP2). Then, the catabolite repression effect was relieved by knocking down the glucose-specific transporter protein (encoded by *ptsG*), after which xylose utilization was enhanced by overexpression of the transcriptional enhancer *xylR*. Finally, the engineered *E. coli* with an introduced glycerol pathway could efficiently and synergistically utilize xylose and glucose to produce 29.4 g/L of 3-HP and 95 g/L of glycerol in fed-batch fermentation [68]. To address the problem of glycerol accumulation, the team proposed two strategies that attenuate glycerol production and enhance glycerol utilization. The strategy of downregulating glycerol production through the modulation of the glycerol synthesis pathway enhanced 3-HP production to 37.6 g/L [69]. The same team also enhanced glycerol utilization by overexpressing endogenous aldehyde dehydrogenase (PuuC), which boosted the 3-HP titer to 53.7 g/L [70]. Similar metabolic engineering strategies were used to construct engineered *C. glutamicum* that produced 3-HP via the glycerol pathway from glucose and xylose, which resulted in a 3-HP titer of 54.8 g/L in fed-batch fermentation [46]. A recent study reported that *K. pneumoniae* can produce 3-HP via the glycerol pathway utilizing glucose as substrate, but it only achieved a low 3-HP titer of 1.77 g/L in fed-batch fermentation [71]. The key to obtaining high titers of 3-HP from sugar via the glycerol pathway appears to be efficient sugar transport and reducing glycerol accumulation.

#### 4.1.2. Biosynthesis of 3-HP via the Malonyl-CoA Pathway

The malonyl-CoA pathway is the most extensively studied biosynthetic pathway when using glucose as a substrate to produce 3-HP, and the highest titer of 3-HP obtained from glucose via the malonyl-CoA pathway (49.04 g/L) [9] was comparable to that obtained via the glycerol pathway (53.7 g/L) [70] in *E. coli*. By regulating the expression of key enzymes in the production pathway, enhancing the supply of acetyl-CoA, and inhibiting the synthesis of fatty acids, the researchers constructed a microbial cell factory that can efficiently produce 3-HP from glucose.

The imbalance between the activity of the two functional domains of MCR, the key enzyme of the malonyl-CoA pathway, is an important factor that limits the production of 3-HP. Liu et al. [72] overexpressed directionally evolved MCR-C and reduced the expression level of MCR-N to bring MCR-N and MCR-C into equilibrium, reaching a 3-HP titer of 40.6 g/L, corresponding to a yield of 0.19 g/g of glucose in fed-batch fermentation. Increasing the activity of ACC, another key enzyme of the malonyl-CoA pathway, is a common strategy to increase the 3-HP titer. Acc1 activity is tightly regulated at the transcriptional level and the protein level in *S. cerevisiae*. Manipulation of the phospholipid synthesis transcriptional regulators to control the transcription level of Acc1 [73] or the use of the phosphorylation-deficient mutant Acc1 [74] are effective strategies for increasing Acc1 activity. Recently, one strategy for the balanced expression of ACC subunits (AccBC and DtsR1) from *C. glutamicum* was developed in *E. coli*, and the engineered strain achieved a maximal productivity of 1.03 g/(L·h) with a high yield (0.246 g/g glucose) and titer (up to 38.13 g/L) through the malonyl-CoA pathway using a cheap carbon source in fed-batch fermentation [75]. 

In order to further increase 3-HP titer, it is necessary to direct carbon flux towards the key precursor acetyl-CoA. Interestingly, an increase in acetyl-CoA flux in yeast was achieved by enhancing the pathway from pyruvate to acetyl-CoA via acetate, rather than by the direct production of acetyl-CoA from pyruvate. Borodina et al. [44] integrated multiple copies of *mcr* from *C. aurantiacus* and a phosphorylation-deficient *acc1* variant into the genome of *S. cerevisiae*, after which the supply of acetyl-CoA was optimized by overexpressing key enzymes (acetyl-CoA synthase (ACS), aldehyde dehydrogenase (ALD6), and pyruvate decarboxylase (Pdc1)) associated with the transformation of acetate into acetyl-CoA. As a result, the strain produced 9.8 g/L of 3-HP from glucose in fed-batch fermentation. Cellobiose was used as a substrate for 3-HP biosynthesis by displaying β-glucosidase (BGL) on the surface of *S. pombe*, which hydrolyzes cellobiose to glucose. In addition, the acetyl-CoA supply was enhanced by the overexpression of endogenous ATD1 (an *S. pombe* homolog of the *S. cerevisiae* ALD6) and ACS from *E. coli* in the engineered strain with an introduced malonyl-CoA pathway, and the engineered strain produced 11.4 g/L of 3-HP in fed-batch fermentation [42]. It is also possible to enhance the supply of acetyl-CoA for 3-HP biosynthesis from glucose by the exogenous addition of acetate [61].

Malonyl-CoA is also a precursor of fatty acid synthesis, but preventing malonyl-CoA spillover by knocking out the fatty acid synthesis pathway is not feasible, as it results in lethality [76]. Researchers have attempted to inhibit fatty acid synthesis by the exogenous addition of cerulenin [77,78], but the high cost of cerulenin makes it unsuitable for the mass production of 3-HP. The genes involved in fatty acid synthesis can be downregulated by antisense RNA technology [76] or the overexpression of the FadR (fatty acid degradation inhibitor) strategy [34], resulting in an enhanced supply of malonyl-CoA in *E. coli*. In addition, a temperature-sensitive fatty acid synthesis mutant, in which the *fabI* gene is normally expressed at 30 °C and is not expressed at 37 to 42 °C, was used to increase the malonyl-CoA precursor pool and, thus, improve the production of 3-HP. The specific productivity of 3-HP in the *E. coli* mutant that overexpressed *mcr*, *pntAB*, and *accABCD* was 2.01 g/gDCW (a 3.8-fold increase) after changing the temperature, and the final titer of 3-HP reached 49.04 g/L in fed-batch fermentation [9].

#### 4.1.3. Biosynthesis of 3-HP via the β-alanine Pathway

Researchers have also investigated the production of 3-HP via the β-alanine pathway, and the process of 3-HP synthesis from glucose or xylose as substrates has been developed using this strategy. Borodina’s team [60] constructed the biosynthetic pathway from β-alanine to 3-HP by overexpressing the optimal enzyme combination of BPAT from *B. cereus* and HPDH from *E. coli*, and the engineered strain produced 13.7 g/L of 3-HP from glucose in fed-batch fermentation. Later, the same team introduced xylose utilization genes in *S. cerevisiae* and then tested the ability to produce 3-HP from glucose or xylose via the β-alanine pathway or the malonyl-CoA pathway. It was shown that the β-alanine pathway was more advantageous in producing 3-HP from xylose, while the malonyl-CoA pathway produced a higher 3-HP titer using glucose as substrate [79]. The key precursor for 3-HP synthesis, β-alanine, can be produced through transamination from oxaloacetate in the anaplerotic pathway, as well as from succinate in the TCA cycle. Song et al. [38] constructed the β-alanine pathway in a high succinate-producing strain of *E. coli* via the overexpression of endogenous aspartate aminotransferase (AspA) and heterologous L-aspartate-α-decarboxylase (PanD) from *C. glutamicum*, and the appropriate enzyme combination to convert β-alanine to 3-HP [80]. The engineered strain produced 31.1 g/L of 3-HP from glucose in fed-batch fermentation. Compared with other 3-HP biosynthesis pathways, the β-alanine pathway has a low titer; therefore, it is urgent to develop enzymes with high catalytic activity which can be used to more efficiently produce 3-HP in the β-alanine pathway.

### 4.2. Microbial Production of 3-HP from Glycerol

Glycerol is the main by-product of the production of biodiesel, as the production of 1 ton of biodiesel is accompanied by the generation of 100 kg of glycerol [54]. As the global demand for biodiesel is continuously increasing, it is urgent to find ways to deal with the crude glycerol derived from biodiesel, ideally through its conversion into high-value-added products. Currently, the research on the production of 3-HP from glycerol is mainly focused on the coenzyme A-dependent pathway and coenzyme A-independent pathway. Diverse host strains, including *K. pneumoniae* [27], *E. coli* [31], *L. reuteri* [39], etc., have been developed as microbial cell factories for 3-HP production via the glycerol pathway and achieved high titers. Recently, a strategy was proposed to develop a *Pichia pastoris* cell factory for 3-HP production from glycerol via the malonyl-CoA pathway.

#### 4.2.1. Biosynthesis of 3-HP via the Coenzyme A-Dependent Pathway

Most studies on 3-HP production via the coenzyme A-dependent pathway were focused on *L. reuteri*, which can naturally metabolize glycerol to produce 3-HP. During the growth of *L. reuteri* on glucose, 3-HPA, which is produced through glycerol dehydration reaction, tends to be reduced to 1,3-PDO rather than oxidized to 3-HP, and only under anaerobic conditions will 3-HPA be converted to equimolar amounts of 3-HP and 1,3-PDO [81]. It has been shown that the conversion of glycerol to 3-HPA catalyzed by glycerol dehydration was 10 times faster than the redox conversion of 3-HPA to 1,3-PDO and 3-HP in *L. reuteri* [15]. As a result, the 3-HPA could not be completely converted and accumulated in the fermentation broth. A co-culture system of wild-type *L. reuteri* and recombinant *E. coli* overexpressing aldehyde dehydrogenase (ALDH) was designed to address this problem. In this system, the 3-HPA produced by *L. reuteri* was further converted to 3-HP by the engineered *E. coli*, and the titers of 3-HP reached 125.93 g/L in modified fed-batch fermentation, respectively [39]. It was reported that recombinant *E. coli* overexpressing PduP, PduL, and PduW from *L. reuteri* produced 3-HP at a yield of 0.68 mol/mol of 3-HPA, without accumulating 1,3-PDO during the production process [82]. Recently, *L. reuteri* was engineered into a whole-cell catalyst with a frozen cross-linked structure to produce 3-HPA and 3-HP at maximum specific reaction rates of 281.4 and 62.4 mg/gCDW/h, respectively [83]. The strategy of immobilized cells reduced production costs and improved production stability.

#### 4.2.2. Biosynthesis of 3-HP via the Coenzyme A-Independent Pathway

The biosynthesis of 3-HP from glycerol via the coenzyme A-independent pathway has been extensively studied. However, it is important to note that imbalances in the activity of key enzymes of the coenzyme A-independent pathway usually lead to the accumulation of the toxic intermediate 3-HPA. Other factors limiting the production of 3-HP from glycerol include the unbalanced growth–production relationship [84] and the availability of coenzyme B_12_. Finally, the conversion of glycerol to 3-HP is accompanied by a net accumulation of reducing equivalents, and it is essential to achieve intracellular redox balance for microorganisms to efficiently metabolize glycerol for the synthesis of 3-HP [85].

Researchers have attempted to enhance the activity of ALDH and balance the production pathway to improve the 3-HP titer obtained by the coenzyme A-independent pathway. A novel aldehyde dehydrogenase (GabD4) from *Cupriavidus necator* was found to have high catalytic activity, and it was modified by site-saturation mutagenesis based on homology modeling. Finally, an engineered *E. coli* expressing a GabD4 mutant with 40% higher enzyme activity produced 71.9 g/L of 3-HP in fed-batch fermentation [32]. To enhance the expression of ALDH, the T7 expression system was introduced into *K. pneumoniae* and the 3-HP yield of the recombinant strain was enhanced by 3.24-fold [86]. Zhao et al. [27] used the tac promoter with three tandem repeats to initiate the transcription of *puuC* (encoding ALDH in *K. pneumoniae*), and the engineered *K. pneumoniae* reached a titer of 102.61 g/L in fed-batch fermentation. However, it is impossible to fully balance the activity of ALDH and GDHt by increasing ALDH activity alone. Regulatory cassettes containing promoters of different strength and double ribosome binding sites were used to express ALDH and GDHt, which showed that 3-HPA accumulation could be completely eliminated when the expression level of ALDH was approximately eight-fold higher than that of GDHt [87]. In a different approach, the catalytic activity of GDHt was precisely downregulated by 5’UTRs engineering, and the yield reached 0.97 g of 3-HP/g glycerol in fed-batch fermentation, which is the highest yield for the production of 3-HP from glycerol reported to date [88].

The main factors limiting the conversion of glycerol to 3-HP are unsuitable glycerol assimilation rates together with unbalanced production and growth relationships. *K. pneumoniae* possesses a thoroughly investigated glycerol metabolic pathway that can rapidly metabolize glycerol. The carbon flux from glycerol towards 3-HP production rather than growth in *K. pneumoniae* has been achieved by decreasing the rate of glycerol assimilation through the deletion of glycerol kinase (GlpK) [89,90]. A tryptophan operon-assisted CRISPR interference (CRISPRi) system that switches the glycerol oxidation and reduction pathways in *K. pneumoniae* has been reported, and the CRISPRi-engineered strain produced 88.8 g/L of 3-HP in fed-batch fermentation [28]. Compared to *K. pneumoniae*, glycerol is utilized less efficiently by *E. coli*. Researchers attempted to knock out *glpK* in *E. coli* to redirect the metabolic flux towards 3-HP production. However, the production of 3-HP did not improve [91]. Interestingly, a significant increase in 3-HP production (from 28.1 g/L to 40.5 g/L) was achieved by overexpressing GlpK and knocking down GlpR (glycerol pathway blocker) to promote glycerol assimilation in *E. coli* [84]. 

It is necessary to overexpress glycerol dehydratase reactivase (GdrAB) to reactivate coenzyme B_12_ for some chassis that cannot synthesize coenzyme B_12_ naturally or produce 3-HP via the glycerol pathway under aerobic conditions. A report showed that oxygen can induce the formation of inactivated coenzyme B_12_, which further causes the inactivation of GDHt by binding tightly to it, resulting in limited flux from glycerol to 3-HP [54]. Lee et al. [31] overexpressed *gdrAB* in engineered *E. coli* by introducing the glycerol biosynthesis pathway from *K. pneumoniae*, and the resulting strain produced 76.2 g/L of 3-HP from pure glycerol and 61 g/L 3-HP from crude glycerol in fed-batch fermentation under aerobic conditions. Similarly, the recombinant *P. denitrificans* also produced 3-HP under aerobic conditions; a poor supply of coenzyme B_12_ was the main factor limiting a high 3-HP titer. The strategy of overexpressing GdrAB was also applied in *P. denitrificans*. The resulting strain produced 102 g/L of 3-HP from pure glycerol and 65 g/L of 3-HP from crude glycerol in fed-batch fermentation [50].

The biosynthesis of 3-HP from glycerol is accompanied by the net accumulation of NADH, and, in order to balance the intracellular reducing power, the strains tend to accumulate large amounts of by-products to consume NADH. The balance of NADH and NAD^+^ is important in the conversion of glycerol to 3-HP. The co-production of 1,3-PDO and 3-HP is a way to achieve a cyclic equilibrium of NAD^+^/NADH, since the reduction of 3-HPA to 1,3-PDO is accompanied by the consumption of NADH [89]. Bioelectrochemical systems (BES) provide a new idea for balancing intracellular reducing power. Some microorganisms can deliver respiratory electrons to solid electrodes under anaerobic conditions. The cellular redox states can be controlled by microbe–electrode interaction via extracellular electron transfer in BES [92]. It has been shown that the use of BES to activate anaerobic respiration to regulate the NADH/NAD^+^ ratio increased 3-HP production by 70% when recombinant *K. pneumoniae* was exposed to an electric field [92]. Niu et al. [93] proposed a strategy for the production of 3-HP from glucose and glycerol as mixed substrates, whereby the metabolism of glucose can consume NADH produced from the metabolism of glycerol.

#### 4.2.3. Biosynthesis of 3-HP via the Malonyl-CoA Pathway

Recently, the potential of *P. pastoris* to produce 3-HP via the malonyl-CoA pathway from glycerol has been reported. Fina et al. [40] increased the expression of *mcr* by a strong constitutive promoter in *P. pastoris*, and then further increased the availability of NADPH and malonyl-CoA. The engineered *P. pastoris* produced 24.75 g/L of 3-HP from glycerol in fed-batch fermentation. Based on this study, the same team further modified the engineered *P. pastoris* to obtain a higher titer of 3-HP by adding a second copy of *mcr-c*, overexpressing Acc1 together with enzymes of the endogenous acetyl-CoA synthesis pathway and deleting genes for by-product formation. This resulted in a 3-HP titer of 37.05 g/L, which is the second highest value achieved in yeast cell factories to date [41]. Therefore, the construction of a malonyl-CoA pathway in strains that can efficiently metabolize glycerol is very promising.

### 4.3. Microbial Production of 3-HP from Acetic Acid, Ethanol, and Fatty Acids

Fatty acids, acetic acid, and ethanol are converted to acetyl-CoA, which is further metabolized for cell growth and product synthesis. Acetyl-CoA is an important precursor for the synthesis of 3-HP, and all three substances are high-quality carbon sources for the production of 3-HP via the malonyl-CoA pathway.

At present, several methods have been developed to convert lignocellulosic biomass and C1 gas into acetate [94]. Recently, acetate has been considered as a potential fermentation substrate, and many studies have attempted to convert acetate into biofuels. However, acetate is less efficiently assimilated by microorganisms than glucose. In a study by Lee et al. [77], acetate consumption was increased by upregulating genes related to acetate assimilation and activating the glyoxalate cycle, and metabolic spillover was prevented by inhibiting the expression of genes related to fatty acid synthesis. Finally, the engineered *E. coli* introducing the malonyl-CoA pathway produced 3 g/L of 3-HP in shake flasks. Similarly, Lai et al. [34] synthesized 15.8 g/L of 3-HP with the yield of 0.71 g/g from acetate, using engineered *E. coli* via the malonyl-CoA pathway. Subsequently, the titer of 3-HP reached 11.2 g/L with the yield of 0.55 g/g when syngas-derived acetic acid was used as a substrate instead of pure acetic acid. Due to the inefficient growth of microorganisms on acetate, a two-stage strategy was developed for the synthesis of 3-HP in engineered *E. coli* that used glucose for cell growth and acetate for 3-HP formation. Finally, the strain produced 7.3 g/L of 3-HP in the two-stage bioreactor [95]. Notably, it is important to balance the TCA cycle and the pathway for 3-HP production, since the whole process from acetate to 3-HP requires the provision of additional reducing power and energy. Chang et al. [47] achieved a balance between the TCA cycle and 3-HP synthesis by regulating the expression of citrate synthase, and the engineered *C. glutamicum* produced the highest titer of 3-HP from acetate reported to date, reaching 17.1 g/L. 

Ethanol can be efficiently synthesized from starch, sugar, lignocellulosic biomass, and C1 gas. Compared to acetate, there are several advantages to utilizing ethanol as a substrate. Instead of consuming one mole of ATP, two moles of NADH are produced when ethanol is assimilated to acetyl-CoA. In addition, the pH of the fermentation broth does not change when ethanol is consumed as a substrate. Recently, it has been demonstrated that *E. coli* has the potential to utilize ethanol to produce 3-HP. Lu et al. [35] increased the titer of 3-HP by optimizing the expression levels of genes in the ethanol utilization pathway and the malonyl-CoA pathway, and explored the effect of deleting or modifying key genes associated with the oxidative branch of the TCA cycle and the gluconeogenesis pathway on 3-HP production. The engineered *E. coli* reached a titer and yield of 13.17 g/L and 0.57 g/g in the whole-cell catalytic system. The low titers of 3-HP from ethanol or acetate as substrates are probably attributed to the fact that these substrates tend to be accumulated as electron acceptors during microbial fermentation, rather than assimilated by microorganisms.

Fatty acids (FAs) are a cheap by-product of the palm oil industry that can effectively provide acetyl-CoA for 3-HP production. Reducing equivalents and ATP are produced during the assimilation of FAs via the β-oxidation pathway. It is worth noting that the theoretical yield of 3-HP from glucose or glycerol was 1 g/g, while from 16-carbon FAs it was 2.49 g/g, which was much higher than from other substrates. To date, the highest 3-HP titer achieved in the malonyl-CoA pathway is 52 g/L, which was produced by an engineered *E. coli* that can efficiently utilize FAs [36]. FAs are a promising substrate for the production of 3-HP via the malonyl-CoA pathway, and the further development of microbial chassis strains that can efficiently utilize FAs is necessary to fully harness this potential.

### 4.4. Microbial Production of 3-HP from 1,3-PDO

In microbial cell factories without knockdown of the 1,3-PDO production pathway (such as engineered *K. pneumoniae* [30,89] and *L. reuteri* [39]), the production of 3-HP via the glycerol pathway is often accompanied by a massive production of 1,3-PDO, exceeding 20 g/L. However, up to four oxidoreductases are involved in the synthesis of 1,3-PDO in *K. pneumoniae*, and it is challenging to completely block the production of 1,3-PDO by gene knockout [96]. Some researchers have even tried to coproduce 1,3-PDO and 3-HP to balance the reducing power [89]. Therefore, it is attractive to further convert 1,3-PDO from fermentation broth into 3-HP. Furthermore, pure 1,3-PDO obtained from the microbial process has a market price of only USD 1/kg [48], which makes 1,3-PDO an economic substrate for 3-HP production. Wild-type *G. oxydans* can naturally catalyze the conversion of 1,3-PDO to 3-HP. Zhao et al. [14] balanced the enzymatic activities of aldehyde dehydrogenase and alcohol dehydrogenase in the 1,3-PDO production pathway by mixing two mutants of *G. oxydans* in a 1:2 ratio, and the resulting artificial consortium converted 40 g/L of 1,3-PDO into 45.8 g/L of 3-HP by whole-cell catalysis. Similarly, endogenous enzymes that convert 1,3-PDO to 3-HP were also identified in *H. bluephagenesis*. An enhanced 1,3-PDO synthesis pathway was constructed by optimizing the expression of endogenous aldehyde dehydrogenase (Aldhb) and endogenous alcohol dehydrogenase (AdhP). The resulting engineered *H. bluephagenesis* produced 154 g/L of 3-HP at the rate of 2.4 g/(L·h) in fed-batch fermentation [27], which was very close to the industrial production requirement. The biosynthesis of 3-HP from 1,3-PDO is a promising strategy but has been less studied, and the construction of a 1,3-PDO pathway in common hosts will likely be an effective way to increase the 3-HP titer.

### 4.5. Microbial Production of 3-HP from Other Carbon Sources

Malonyl-CoA can be produced directly from malonic acid by malonyl-CoA synthase (MatB); subsequently, malonyl-CoA is converted to 3-HP by MCR. The biosynthetic pathway from malonyl acid to 3-HP is shorter compared to other carbon sources [37]. However, the engineered *E. coli* produced only 1.20 g/L of 3-HP from malonic acid in shake flasks [37]. In addition, 3-hydroxypropionitrile has also been developed as a substrate for the production of 3-HP. It was reported that *Meyerozyma guilliermondii* containing nitrile hydrolase completely hydrolyzed 3-HPN into 3 HP with a titer of 19.48 g/L [97]. To mitigate global warming, third-generation (3G) biorefineries aim to produce high-value chemicals and biofuels from greenhouse gases or CO_2_-derived syngas. Lee et al. [53] designed a type II methanotrophic cell factory based on *Methylosinus trichosporium* OB3b to produce 3-HP from CO_2_ and CH_4_ via the malonyl-CoA pathway, but the 3-HP titer reached only 60.59 mg/L in a bioreactor. The feasibility of using microalgae to convert CO_2_ to 3-HP was demonstrated by constructing engineered *Synechococcus elongatus* PCC 7942 with a malonyl-CoA pathway and β-alanine pathway. The strain produced 665 mg/L of 3-HP via the malonyl-CoA pathway and 186 mg/L of 3-HP via the β-alanine pathway [98]. The ability of microorganisms to produce high-value-added chemicals from one-carbon substrates is limited by factors such as carbon fixation efficiency and metabolic properties, and the production capacity of 3G biorefineries for synthesizing 3-HP remains low.

## 5. Fermentation Processes

### 5.1. Fed-Batch Fermentation

The microbial synthesis of 3-HP was mainly developed in laboratory-scale bioreactors using fed-batch fermentation. In these processes, the concentration of substrate is usually kept low, which prevents high substrate concentrations from inducing the production of by-products and inhibiting microbial growth [99]. Among the key enzymes commonly selected for the construction of the glycerol pathway in engineered strains, the activity of GDHt is higher than that of ALDH, and the accumulation of toxic intermediate 3-HPA can be prevented by limiting the glycerol concentration. *K. pneumoniae* was found to be subject to significant substrate inhibition when the glycerol concentration was increased to above 40 g/L under anaerobic conditions [100]. Acetate must be maintained at a low concentration when used as substrate for 3-HP synthesis because a high concentration of acetate is toxic to cells. According to Chang et al. [47], the initial concentration of acetate was only 1.4 g/L in fed-batch fermentation for the synthesis of 3-HP from acetate by engineered *E. coli*. A sugar-limited fed-batch fermentation that maintains basal levels of glucose and xylose in the medium was designed for 3-HP synthesis by engineered *E. coli* via the glycerol pathway, because high concentrations of glucose and xylose would prevent the efficient conversion of glycerol to 3-HP [69].

In addition to feeding strategies based on the substrate concentration, researchers have also developed alternative feeding strategies for 3-HP production. Lee et al. [31] selected the pH-stat feeding strategy guided by pH in the production of 3-HP, which employs automated feeding when the pH rises above 7.02. To mitigate the metabolic stress response, a recirculating feeding method was developed for 3-HP production, in which the fermentation broth was centrifuged, mixed with fresh medium, and finally, returned to the original bioreactor [28]. 

### 5.2. Two-Stage Culture Process

Two-stage culture processes have been developed to better balance growth and production. This is generally achieved either by using different carbon sources or by setting different parameters during the fermentation process. In studies on 3-HP production from 1,3-PDO using *H. bluephagenesis*, glucose was used as the carbon source during the growth phase, while during the production phase, the concentration of glucose was maintained at 5 to 10 g/L for cell growth, and 1,3-PDO was added to produce 3-HP [48]. The recombinant *E. coli* with weakened GlpK, which synthesized 3-HP from glycerol, could not perform well for growth. Therefore, 20 g/L of glucose was initially added for well-grown cells; then, 57–60 g/L of glycerol was added for 3-HP production during the fermentation process [101]. Acetate is a high-quality substrate for the synthesis of 3-HP via the malonyl-CoA pathway, yet it is not a good substrate for cells growth in some strains. Lama et al. [95] constructed an engineered *E. coli* to first rapidly assimilate glucose for cell growth at a high aeration rate of 2 vvm, and then to use acetate as a carbon source for 3-HP production at a low aeration rate of 0.5 vvm. Since the optimal 3-HP production condition for *K. pneumoniae* was microaerobic condition, there was a study on the 3-HP production from glycerol by engineered *K. pneumoniae* employing a two-stage aeration strategy, in which cell growth was performed under high aeration conditions and 3-HP production was performed under aeration rates reduced by half [102]. 

### 5.3. Modular Processes

The modular process strategy has been successfully applied to improve the yield of 3-HP by splitting the 3-HP biosynthetic pathway into several simple modules, and then assigning specific modules to suitable strains, finally, integrating the individual modules to produce 3-HP. According to Sabet-Azad et al. [82], *L. reuteri* was able to efficiently convert glycerol to 3-HPA and some 3-HP in fed-batch fermentation. Then, 3-HPA was recovered from the fermentation broth by complexation with bisulfite, and *E. coli* was introduced into the production module to catalyze the conversion of 3-HPA to 3-HP at a yield of 1 mol/mol in the whole-cell catalytic system. In contrast to the previous process that used two modules in separate stages, a study attempted to co-culture *L. reuteri* and recombinant *E. coli* at appropriate ratios, and the system produced 125.93 g/L of 3-HP and 88.46 g/L of 1,3-PDO through whole-cell catalysis [39]. Another modular production strategy was developed by Zhao et al. [30]: glycerol was converted to 1,3-PDO by *K. pneumoniae* in fed-batch fermentation, after which *G. oxydans* DSM2003 converted 1,3-PDO to 3-HP through whole-cell biocatalysis. Eventually, this microbial system produced 242 g of 3-HP from 480 g of glycerol in a 7.0 L bioreactor with a yield and titer of 0.5 g/g and 60.5 g/L, respectively. This modular production strategy effectively utilized the advantages of different strains in 3-HP synthesis, which improved the yield of 3-HP but increased the production cost due to the complexity of the 3-HP production process. At present, the reported 3-HP module process involves the collection and washing of biomass from a second module or two modules.

## 6. Conclusions and Future Prospects

In recent years, a large number of studies have achieved the efficient conversion of renewable substrates into 3-HP using engineered microorganisms. As outlined in this paper, the design of microbial cell factories that can efficiently produce 3-HP is determined by selecting an appropriate biosynthetic pathway, production chassis, and high-quality carbon source. With continuing research on the microbial synthesis of 3-HP, the range of substrates that can be used by microbial cell factories has been gradually increased. Initially, most studies used glycerol and glucose as substrates, after which lignocellulose, acetate, ethanol, fatty acids, and other alternative substrates were also used for 3-HP synthesis. Researchers are increasingly using renewable feedstocks that are cheaper, easier to obtain, and more environmentally friendly. Combining all reports on the microbial production of 3-HP, a conclusion was drawn that the fewer catalytic reactions required to move from substrate to product, the easier it seems to be to obtain high titers of 3-HP. For each 3-HP production pathway that has been developed, there should be one or more suitable carbon sources (inexpensive, converts to 3-HP by fewer reactions, and can be rapidly assimilated) corresponding to it. There is no doubt that glycerol is the most suitable carbon source for the glycerol pathway, and high 3-HP titers have been achieved in *K. pneumoniae* and *P. denitrificans* that are able to naturally and rapidly metabolize glycerol. In particular, the 3-HP productivity of the *P. denitrifiers* cell factory was excellent, reaching 2.5 g/(L·h) (which is the minimum requirement for the microbial 3-HP production to be economically competitive) [50]. Reports on the 3-HP production by recombinant *P. denitrifiers* are scarce, and the production process needs to be further explored. For the malonyl-CoA pathway, it is more appropriate to use substrates that can be efficiently converted to acetyl-CoA, such as acetate and fatty acids. The synthesis of 3-HP from the above substrates is mainly performed in *E. coli* due to its broad substrate spectrum and clear genetic background. The assimilation of acetate or fatty acids by *E. coli* still needs to be further strengthened. The β-alanine pathway seems to be less competitive compared to the two pathways mentioned above because of the longer reaction route. A simplified pathway, the oxaloacetate pathway, was recently explored in *S. cerevisiae*, which showed excellent 3-HP production ability. It is worth testing the effects of the oxaloacetate pathway in other chassis. Finding an inexpensive carbon source that can be efficiently converted to oxaloacetate in a few steps is also essential in the future. In addition, engineered strains need to be matched with appropriate fermentation processes to avoid the accumulation of by-products and to balance production and growth. Dynamic regulation devices have also been developed to address these issues, and good 3-HP titers were achieved in each study [9,28]. Designing or modifying dynamic conditioning devices is an effective strategy to improve the efficiency of engineered strains in the future.

Third-generation microbial refineries that synthesize 3-HP directly from CO_2_ have been successfully constructed, but the titers are currently at the mg/L level. Further modification of the third-generation microbial refineries for the synthesis of 3-HP to achieve high titers is necessary to unlock the future prospects of environmentally friendly and sustainable production. The efficiency of photosynthesis plays a decisive role in the productivity of cell factories based on cyanobacteria. In the future, it will become possible to optimize the components and pathways of the cyanobacterial photosynthetic system to enhance carbon uptake, sequester carbon, and reduce carbon loss [103]. 

## Figures and Tables

**Table 1:** Representative studies on biotechnological 3-HP production using different chassis strains from various carbon sources.

Microorganisms	Carbon Source	Pathway	Engineering Strategy	Titer (g/L)	Productivity (g/(L·h))	Yield (g/g)	Ref.
*K. pneumoniae*	Glycerol	Glycerol oxidation via the coenzyme A-independent pathway	Expression of *puuC*, using tandem repeat tac promoter	102.6	1.07	0.86	[27]
*K. pneumoniae*	Glycerol	Glycerol oxidation via the coenzyme A-independent pathway	Development of a CRISPRi system to switch glycerol oxidation and reduction pathways	88.8	0.98	-	[28]
*K. pneumoniae*	Glycerol	Glycerol oxidation via the coenzyme A-independent pathway	Expression of *puuC*, using tac promoter and deletion of *pta*, *ldhA*	83.8	1.16	0.59	[29]
*K. pneumoniae* and *G. oxydans*	Glycerol	Glycerol oxidation via the coenzyme A-independent pathway	Development of a two-step process	60.5	1.12	0.50 *	[30]
*E. coli*	Glycerol or Crude glycerol	Glycerol oxidation via the coenzyme A-independent pathway	Expression of *dhaB1234*, *gdrAB*, *ydcW*; control of the dissolved oxygen (DO) level	76.2 or 61	1.89 or 2.28	0.45 or 0.594	[31]
*E. coli*	Glucose and glycerol	Glycerol oxidation via the coenzyme A-independent pathway	Expression of gabD4; knocking out of *ackA*-*pta*, *yqhD*	71.9	1.80	- **	[32]
*E. coli*	Glycerol	Glycerol oxidation via the coenzyme A-independent pathway	Adaptive laboratory evolution resulted in a highly 3-HP-tolerant strain	63.05	-	-	[33]
*E. coli*	Glucose	Malonyl-CoA pathway	Expression of *mcr*, *accABCD*, *pntAB*; deletion of *ldhA*, *pflB*, *poxB*, *pta*, *ackA*, *mgsA*, *fabI*	49.04	0.71	-	[9]
*E. coli*	Acetate	Malonyl-CoA pathway	Expression of *acc*, *mcr*, *ack-pta*; dynamic regulation of the expression of *sdh*; deletion of *fadR*	15.8	0.39	0.71 *	[34]
*E. coli*	Ethanol	Malonyl-CoA pathway	Expression of mcr, *adhE*, *icd*, and *pntAB*; deletion of *gcl* and *fadR*	13.1	0.14	0.57 *	[35]
*E. coli*	Fatty acids	Malonyl-CoA pathway	Expression of *atoSC*, *fadD*, *fadL*, and *PntAB*; optimized expression of *mcr*; deletion of fadR	52	1.13	1.56 **	[36]
*E. coli*	Malonate	Malonyl-CoA pathway	Expression of *matB*, *mcr*, *pntAB*, and *yfjB*	1.2	-	- **	[37]
*E. coli*	Glucose	β-alanine pathway	Expression of *pa0132*, *ydfG*, *panD*, *aspA*, *ppc*; deletion of *fumAC*, *fumB*, *iclR*	31.1	0.63	0.423	[38]
*L. reuteri *and *E. coli*	Glycerol	Glycerol oxidation via the coenzyme A-dependent	Expression of *gabD4* and *pduQ*; concurrent production	125.93	2.47	0.95 *	[39]
*P. pastoris*	Glycerol	Malonyl-CoA pathway	Expression of *mcr*, *acc*, and *cPOS5*	24.75	0.54	0.13	[40]
*P. pastoris*	Glycerol	Malonyl-CoA pathway	Expression of *mcr*, *acc1*, *acs*, *ald6*, *pdc1*; deletion of *ArDH*	37.05	0.71	0.18	[41]
*S. pombe*	Cellobiose	Malonyl-CoA pathway	Expression of *mcr*, *acc*, *BGL*, *acs*, and *atd1*; deletion of *adh*	11.4	0.14	0.11	[42]
*D. hansenii*	Glucose and propionic acid	-	Screening from orchard soil and human excrement as raw material	62.42	1.30	-	[43]
*S. cerevisiae*	Glucose	Malonyl-CoA pathway	Expression of *mcr*, *ALD6*, *pdc1*, *acc1*, *acs*, *GAPDH*	9.8	0.09	0.06	[44]
*S. cerevisiae*	Glucose	β-alanine pathway	Expression of *pyc1*, *pyc2*, *ATT2*, *panD*, *Bapat*, and *ydfG*; optimization of culture conditions	about 25	-	0.25	[45]
*S. cerevisiae*	Glucose	Oxaloacetate pathway	Expression of *pyc*, *mdlC*, *mmsB*, *glc7*, *ptc7*	18.1	0.17	0.125	[26]
*C. glutamicum*	Glucose or (Glucose and xylose)	Glycerol oxidation via the coenzyme A-independent pathway	Expression of *gpd* and *gpp*, *pduCDEGH*, *araE* and *xylAB*; deletion of *ldhA*, *pta-ackA*, *poxB*, *glpK*	62.6 or 54.8	0.86 or 0.76	0.51 or 0.49 **	[46]
*C. glutamicum*	Acetate	Malonyl-CoA pathway	Optimization of *mcr* and attenuation of *gltA*	17.1	0.14	0.10	[47]
*H. bluephagenesis*	1,3-Propanediol	1,3-Propanediol pathway	Expression of *dhaT* and *aldD*	154	2.4	0.93 **	[48]
*G. oxydans*	Glucose and 1,3-Propanediol	1,3-Propanediol pathway	Expression of *ADH* and *ALDH*	45.8	1.86	1.14 *	[24]
*B. subtilis*	Glycerol	Glycerol oxidation via the coenzyme A-independent pathway	Expression of *dhaB* and *puuC*; deletion of *glpK*	10	-	0.79	[49]
*P. denitrificans*	Glycerol or Crude glycerol	Glycerol oxidation via the coenzyme A-independent pathway	Expression of *dhaB*, *gdrAB* and *kgsadh*; deletion of *3hpdh, 3hibdh;* replacement of coenzyme B_12_ pathway	102 or 65	2.5 or -	-	[50]
*P. asiatica*	Glucose and glycerol	Glycerol oxidation via the coenzyme A-independent pathway	Expression of *dhaB*, *gdrAB* and *kgsA*; deletion of *glpR*, *glpK* and *crc*	63	2.15	0.96 **	[51]
*Synechocystis* sp*. PCC 6803*	CO_2_	Malonyl-CoA pathway	Expression of *mcr*, *accBCAD*, *birA*, *pndAB*, *rbc*; deletion of *pta*, *phaB*	0.83	-	-	[52]
*type II methanotroph*	CH_4_	Malonyl-CoA pathway	Expression of *mcr*, *acc*, *BPL*, *ME*, and *mmc*	0.60	-	0.02	[53]

* Indicates that 3-HP was produced through whole-cell catalysis, ** indicates that the study used different carbon sources during the growth and production stages, and the yield was mainly for the production stage. Genes: *3hibdh*: 3-hydroxyisobutyrate dehydrogenase; *3hpdh*: 3-hydroxypropionate dehydrogenase; *AAT2*: aspartate aminotransferase; *acc1*/*accABCD*/*acc*: acetyl-CoA carboxylase; *ackA*: acetate kinase; *acs*: acetyl-CoA synthase; *ADH*/*adhE*: alcohol dehydrogenase; *ald6*/*aldD*/*ALDH*: aldehyde dehydrogenase; *aspA*: aspartase; *araE*: arabinose transporter; *ArDH*: D-arabitol dehydrogenase; *bapat:* aminotransferase; *BGL*: displaying β-glucosidase; *birA*: biotinilase; *BPL*: biotin protein ligase; *cPOS5*: cytosolic NADH kinase; *crc*: the global regulatory protein; *dhaB*: glycerol dehydratase; *dhaT*: 1,3-propanediol dehydrogenase; *fabI*: enoyl-acyl carrier protein; *fadR*: fatty acid degradation inhibitor; *fumAC*: fumarase A and C; *fumB*: fumarase B; *gabD4*: aldehyde dehydrogenase; *GAPDH*: glyceraldehyde-3-phosphate dehydrogenase; gcl: tartronate-semialdehyde synthase; *gdrAB*: glycerol dehydratase reactivase; *glc7*: protein phosphatase regulator GIP1; *glpK*: glycerol kinase; *glpR*: glycerol catabolic pathway repressor; *gltA*: citrate synthase; *gpd*: glycerol 3-phosphate dehydrogenase; *gpp*: glycerol 3-phosphate phosphatase; *Icd*: isocitrate dehydrogenase; *kgsA*: 16S rRNA (adenine1518-N6/adenine1519-N6)-dimethyltransferase; *kgsadh*: α-ketoglutaric semialdehyde dehydrogenase; *ldhA*: lactate dehydrogenase; *mcr*: malonyl-CoA reductase; *mdlC*: benzoylformate decarboxylase; *ME*: NADP-dependent malic enzyme; *mmc*: methylmalonyl-CoA carboxyltransferase; *mgsA*: methylglyoxal synthase; *mmsB*:3-hydroxyisobutyrate dehydrogenase; *pa0132:* β-alanine pyruvate transaminase; *panD*: aspartate 1-decarboxylase; *pdc1*: pyruvate decarboxylase; *pduCDEGH*: diol dehydrogenase; *pduQ*: 1,3-propanediol oxidoreductase; *pflB*: pyruvate formate-lyase; *phaB*: acetoacetyl-CoA reductase; *pntAB*: pyridine nucleotide transhydrogenase subunits α and β; *poxB:* pyruvate oxidase; *ppc:* phosphoenolpyruvate carboxylase; *ptc7*: type 2C serine/threonine protein phosphatase; *pyc1*/*pyc2*/*pyc*: pyruvate carboxylases; *pta*: phosphate acetyltransferase; *puuC*: aldehyde dehydrogenase; *rbc*: ribulose bisphosphate carboxylase oxygenase (Rubisco); *sdh*/*sdhC:* succinate:quinone oxidoreductase; *xylA*: xylose isomerase; *xylB*: xylulokinase; *xylR*: DNA-binding transcriptional dual regulator; *ydcW*: γ-aminobutyraldehyde dehydrogenase; *ydfG:* 3-hydroxy acid dehydrogenase*; yqhD*: NADPH-dependent aldehyde reductase.

## Data Availability

No new data created.
